# MySSP: Non-stationary evolutionary sequence simulation, including indels

**Published:** 2007-02-26

**Authors:** Michael S. Rosenberg

**Affiliations:** Center for Evolutionary Functional Genomics and the School of Life Sciences, Arizona State University, Tempe, AZ, USA

**Keywords:** Sequence Simulation, DNA, Indels, Non-stationarity

## Abstract

MySSP is a new program for the simulation of DNA sequence evolution across a phylogenetic tree. Although many programs are available for sequence simulation, MySSP is unique in its inclusion of indels, flexibility in allowing for non-stationary patterns, and output of ancestral sequences. Some of these features can individually be found in existing programs, but have not all have been previously available in a single package.

## Introduction

Simulation of molecular sequence evolution has become a fundamental part of comparative genomic and bioinformatics analysis. Simulation has proven particularly useful for testing the efficacy of bioinformatics methods and techniques under a variety of conditions and assumptions (or violations thereof), including, for example, phylogenetic analysis (Hillis 1995; Nei 1996; Takahashi and Nei 2000; Rosenberg and Kumar 2003; Huelsenbeck and Rannala 2004, just to name a few) and sequence alignment (Keightley and Johnson 2004; Pollard et al 2004; Rosenberg 2005). Many programs are available for simulating molecular sequence evolution, including Evolver (PAML) (Yang 1997), Seq-Gen (Rambaut and Grassly 1997), ROSE (Stoye et al 1998), and DAWG (Cartwright 2005), each with its own set of strengths and weaknesses. The program presented here, MySSP, has been gradually developed over a series of projects (including, eg, Rosenberg and Kumar 2001; Rosenberg and Kumar 2003; Gadagkar et al 2005; Rosenberg 2005) and is being made publicly available because of some unique features, individually and in combination, which are not found in other available packages.

As with many similar programs, given a fixed tree (supplied by the user) MySSP constructs an initial DNA sequence at the root of the tree and simulates evolution across the tree using a variety of common models of DNA evolution, including Jukes-Cantor (Jukes and Cantor 1969), Kimura two-parameter (Kimura 1980), equal input, Hasegawa-Kishino-Yano (Hasegawa et al 1985), and the general time-reversible model. Rate variation among sites can optionally be modeled with the standard gamma-distribution for any of these models. Multiple genes with different parameters and models can be simulated simultaneously. MySSP is designed for large-scale studies, including simulation of multiple replicates and outputs sequences into NEXUS, MEGA, or FASTA formats. MySSP has a fairly simple GUI for basic use, but also has a specialized batch script interpreter to allow for more complicated or large-scale simulations.

Where MySSP becomes unique relative to most other simulation programs is (1) its ability to simulate insertion and deletion events; (2) its ability to allow simulation of nonstationary processes and models across the tree; and (3) its option to output ancestral sequences. Two of these features (1 and 3) can individually be found in existing programs, but not all have been previously available in a single package. Each is described in turn.

## Simulation of Insertions and Deletions

Insertions and deletions (indels) are a common component of sequent evolution, but historically have not been included in most simulation packages; only two are known to include indel evolution: ROSE (Stoye et al 1998) and DAWG (Cartwright 2005). MySSP simulates insertions and deletions using simple Poisson models for rate and size distribution of insertion and deletion events (modeled separately, parameters provided by the user). One advantage of MySSP is that the output sequences are aligned correctly, ie, the output sequences include gaps such that aligned sites across sequences represent true homologies. This gives one a baseline “true alignment” that can be used to contrast with the results from removing the gaps from the output sequences (a trivial exercise) and running them through a standard alignment program.

## Non-stationary processes and models

A common concern in molecular sequence analysis is whether the evolutionary process is stationary across a tree. While there are many possible models of sequence evolution, the majority of simulation programs assume that whatever model is specified is constant throughout the tree. MySSP allows the user to change the evolutionary model for each and every branch, if they desire. One can completely change every aspect of the model, including basic substitution pattern (JC, HKY, etc.), transition-transversion bias, gamma distributed rate variation, equilibrium nucleotide frequencies, and indel rate and size. One can also change the basic rate of substitution for a branch, increasing or decreasing it relative to that found on the model tree. This flexibility allows one to much more easily examine the effects of non-stationary processes on bioinformatics analysis, eg, using a single “average” model in maximum likelihood phylogenetic analysis. The ability to completely change the model for each and every aspect of the tree is unique among simulation programs.

## Ancestral sequences

MySSP also includes an option for outputting ancestral sequences, that is, the sequence found at each and every node on the tree. This may be useful for those wishing to test methods of ancestral state reconstruction or for whom tracing changes from ancestral sequences may be important. Ancestral sequence output is available from Evolver (Yang 1997) and Seq-Gen (Rambaut and Grassly 1997), but not in combination with indel and non-stationary simulation.

## Availability

The program and documentation can be freely downloaded from http://lsweb.la.asu.edu/rosenberg. It runs natively under all 32-bit Windows operating systems and has also successfully been used under Linux emulators. Source code is available on request.
